# Health Literacy, Covid-19 and risk perception: a cross-sectional survey in Prato in the 2nd wave

**DOI:** 10.1093/eurpub/ckac131.362

**Published:** 2022-10-25

**Authors:** B Velpini, E Stancanelli, L Stacchini, M Bruschi, V Lastrucci, F Puggelli, R Berti, C Lorini, G Bonaccorsi

**Affiliations:** 1 Department of Health Sciences, University of Florence, Florence, Italy; 2 Epidemiology Unit, Meyer Children’s University Hospital, Florence, Italy; 3 Management Department, Meyer Children’s University Hospital, Florence, Italy; 4 Department of Prevention, Azienda Sanitaria Locale Toscana Centro, Florence, Italy

## Abstract

During the Covid-19 pandemic, individual and collective public health measures were undertaken to control the spread of the virus. Their effectiveness relies on people’s abilities to understand and adopt the correct behaviors. This study aims to evaluate the role of Health Literacy (HL) in influencing the adherence to Covid-19 preventive measures and risk perception of a sample of workers employed in various activities involving close contact with the population in the province of Prato (Tuscany, Italy) in the second pandemic wave (November-December 2020). A cross-sectional survey was conducted on a sample of public workers (e.g., teachers, educators, assistants/aides, other health personnel). Data on knowledge, attitudes and practices towards (KAP) Covid-19 preventive measures and risk perception were collected. HL was measured with the HLS-EU-Q6 tool. Spearman correlation analysis was used to assess the correlation between HL and KAP and Covid-19 risk perception. Multivariate linear regression analyses were performed to evaluate the role of HL in predicting KAP and Covid-19 risk perception, adjusted for sex, age, comorbidity, educational level, country of birth. A total of 402 people participated in this study; 47.8% had a problematic HL level. The HL level was correlated with KAP and practices towards Covid-19 prevention measures; no significant associations were found with Covid-19 risk perception. In multivariate models, HL significantly and positively predicted a higher level of knowledge of Covid-19 preventive measures (B = 0.413 for problematic HL; B = 0.542 for sufficient HL). Confirming a previous study conducted in Prato in the first pandemic wave, HL did not predict adherence to Covid-19 infection control measures, probably due to fear of the disease and attention towards prevention behaviors being still higher in the second pandemic wave.

**Key messages:**

• HL skills are linked to understanding of public health measures.

• HL skills should be improved to favor the adherence to correct behaviors.

